# Culturally Responsive Psychiatric Services for Refugee and Immigrant Adolescents: Are Child and Adolescent Psychiatrists Prepared to Serve Refugee Children? A Focus on African Refugee Families

**DOI:** 10.1089/heq.2020.0117

**Published:** 2021-05-13

**Authors:** Rhonda BeLue, Alicia Barnes, Sunita Manu, Camille Luckett, Balkozar Adam

**Affiliations:** ^1^ Department of Health Management and Policy, College of Public Health and Social Justice, Saint Louis University, St. Louis, Missouri, USA.; ^2^ Department of Psychiatry and Behavioral Neuroscience, Saint Louis University, St Louis, Missouri, USA.; ^3^ Department of Psychiatry, University of Missouri-Columbia, Columbia, Missouri, USA.

**Keywords:** psychiatric services, African immigrants and refugees, mental health

## Abstract

The arrival of sub-Saharan African immigrants and refugees (AIRs) to the United States has been steadily increasing for the past several decades. Not only are AIR adolescents directly affected by previous migration processes, but they are also impacted by stress and the mental health of their parents, even if they were born in the United States to immigrant/refugee parents. Immigrant and refugee parents concerned with their child's behavior and emotions should be evaluated by a qualified mental health professional, including licensed counselors, psychologists, and child and adolescent psychiatrists. However, access to culturally responsive psychiatric care for youth is limited. African adolescents are additionally burdened by their own acculturation process, balancing multiple cultural expectations as well as feelings of social isolation resulting from perceived racism and discrimination.

The arrival of sub-Saharan African refugees to the United States has been steadily increasing for the past several decades. What makes African immigrants and refugees (AIRs) especially vulnerable to mental health challenges is that many aspects of their new lifehousing options, employment opportunities, health care access, social networks, and cultural normsare dramatically different from those in their countries and can be challenging to negotiate. AIRs often suffer from stress and emotional health issues.^1^ There is also evidence that AIRs are at increased risk for suicide due to acculturation and the challenges inherent in accessing social services.^2^ Not only are adolescents directly affected by previous migration processes, but they are also impacted by stress and the mental health of their parents, even if they were born in the United States to immigrant/refugee parents.^3^ Hence, the trauma experienced before migration and the acculturative process experienced by parents can have an ongoing and intergenerational effect on children born postmigration.^4^ African adolescents are additionally burdened by their own acculturation process, balancing multiple cultural expectations as well as feelings of social isolation resulting from perceived racism and discrimination.^5^

Acculturative and premigration stress can have a significant effect on AIR adolescents, including both pre- and postmigration stress such as exposure to war, parental mental health problems, potential discrimination, and lack of peer and family support in the receiving country.^6^

Refugee youth are learning to balance two or more cultures. Teenagers who only have one culture struggle to fit in or feel accepted. Refugees are dealing with all that plus language barriers, financial obstacles, questions about their culture, and worthiness of their identity. Intergenerational acculturation stress is often observed in these families headed by parents with traditional cultural beliefs.^7^

Recent studies suggest that mental health services for refugees can be made more culturally responsive by training mental health providers in the delivery of culturally responsive care, developing partnerships that facilitate financial and physical access,^8^ and working with language interpreters to improve the delivery of care that accounts for cultural and social experiences.^9^

Immigrant and refugee parents concerned with their child's behavior and emotions should be evaluated by a qualified mental health professional, including licensed counselors, psychologists, and child and adolescent psychiatrists. However, access to culturally responsive psychiatric care for youth is limited. According to the American Academy of Child and Adolescent Psychiatry (2018), there is a severe shortage of child and adolescent psychiatrists in the United States. Although close to one in five youth have mental, emotional, or behavioral disorders annually, of those only 20% received treatment from a mental health provider. This issue becomes more acute for refugee children given that they already have limited access to care compared with their U.S. born counterparts. Mental health services for immigrants and refugees such as AIRs are most effective when culturally responsive, yet there is no widespread systematic approach to establishing culturally responsive care across various mental health care settings.^10^

This region is the home of significant Congolese, Liberian, Sierra Leonean, Ogoni-Nigerian immigrant, and refugee communities. Participants were asked about their experience, training, and needs related to providing services to AIR children. Participant responses were recorded and transcribed verbatim. All procedures were approved by the Institutional Review Board of the authors' institution.

A listening session with a mid-western child and adolescent psychiatric professional society (*n*=8) practicing psychiatrists from diverse settings was queried during the organization's quarterly meeting in September 2020. Participants were recruited through a study team member (A.B.) who is a member of the psychiatric professional society. A.B. arranged for our study team to present information about our project followed by a listening session at the Fall 2020 quarterly meeting at the psychiatric professional society. Participation in the listening session was open to any society member who wanted to attend. Listening sessions are similar to focus groups^11^; however, listening sessions are less structured. Focus groups may have a more formal structure and interview guide, whereas listening sessions allow for participants to express their ideas, opinions, and beliefs about a topic. In our case, participants were prompted to express their experience with providing care for AIRs.

Participants discussed (1) current emotional and mental health challenges among African and other refugee children as experienced by child and adolescent psychiatrists, (2) challenges specific to treatment for African origin immigrants and refugee youth, and (3) educational and clinical structural improvements needed for psychiatrists to provide culturally responsive care to AIR children and adolescents.

Please see key issues highlighted by listening session participants in Figure 1.

**FIG. 1.**
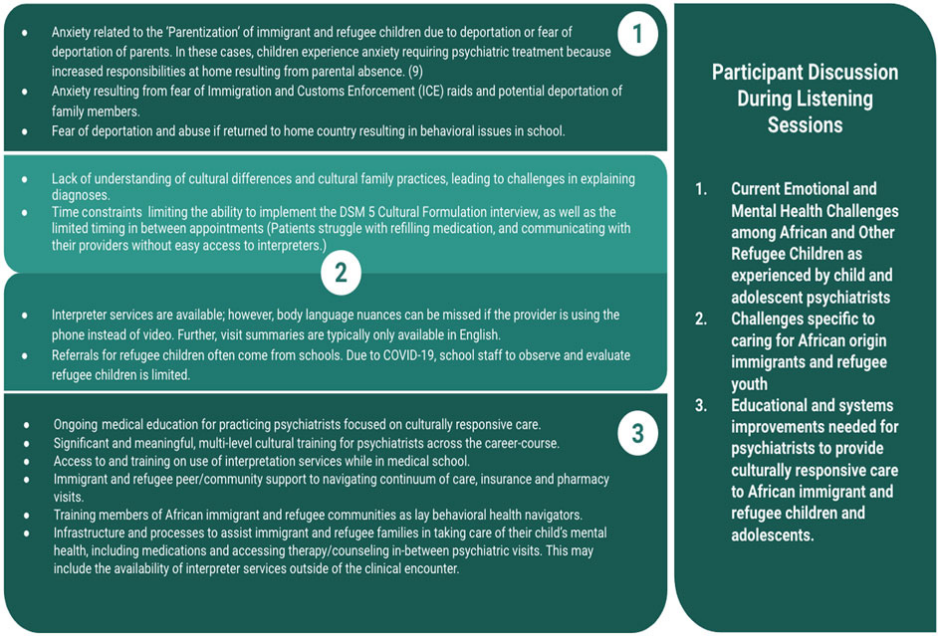
Key issues highlighted by listening session participants.

## Conclusion

Refugees may be reluctant to receive mental health care due to distrust of authority and of the system of care. The stigma of mental illness and the fear of being labeled crazy may mean mental illness are kept secret. Having an interpreter/translator who speaks the language and the dialect, who the family would accept, could be challenging to find.^12^ The families may be overwhelmed with the migration experience. Although they may identify the presence of emotional problems, it may not be a priority to address. In addition, there may be a fundamental difference in beliefs about mental illness between the refugee patient and the provider. Culturally diverse families often prefer ethnically specific community clinics or clinics located within their communities.^13^

In sum, practicing psychiatrists feel that they are not prepared to provide culturally responsive psychiatric care to AIR adolescents and children. Changes in behavioral health care services delivery are necessary to improve care, including longer appointment times to facilitate the implementation of the DSM 5 cultural formulation interview, availability of interpreter services outside of the clinical encounter, and early and ongoing cultural education for practicing psychiatrists and psychiatrists in training.

## Abbreviation Used

AIR: African immigrant and refugee
